# Problematic social media use and self-rated health among Swedish adolescents: is the association moderated by family support?

**DOI:** 10.1186/s12889-025-22927-6

**Published:** 2025-05-06

**Authors:** Åsa Ledel, Sara Brolin Låftman, Jonas Landberg

**Affiliations:** Department of Public Health Sciences, Stockholm University, Stockholm, SE-106 91 Sweden

**Keywords:** Social media, Problematic social media use, Self-Rated health, Family support, Adolescents, HBSC, Sweden

## Abstract

**Background:**

Recent studies have documented a range of adverse health outcomes associated with excessive social media use among adolescents. Fewer studies have examined potential protective factors in this context. Our study aims to bridge this gap by exploring the relationship between Problematic Social Media Use (PSMU) and poor Self-Reported Health (SRH) among Swedish adolescents, while also examining the potential buffering role of family support.

**Methods:**

The data was sourced from the 2017/18 cross-sectional Swedish Health Behaviour in School-aged Children (HBSC) study, encompassing 3,135 students aged 11, 13, and 15 years. PSMU was measured using the Social Media Disorder Scale and categorized into three levels: low risk of PSMU, moderate risk of PSMU and having PSMU. Poor SRH was defined as a binary variable, with ‘Less than good health’ as the outcome category. Family support was measured using three items that quantified the level of emotional support received, categorized into an index of low, moderate, and high family support. Logistic regression analyses were performed to examine the association between PSMU and poor SRH, adjusting for age, gender, and family affluence. The potential moderating effect of family support was analyzed by including variables to test for both multiplicative and additive interaction between PSMU and family support on the risk of poor SRH.

**Results:**

After adjustment for covariates, the results revealed a graded association between PSMU and poor SRH, with adolescents classified as having PSMU showing the highest odds of poor SRH, followed by those at moderate risk for PSMU. No statistically significant interaction was found on the multiplicative scale. The additive interaction analysis indicated potential trends of a buffering effect of high family support on the risk of poor SRH among adolescents with PSMU, although this was not statistically confirmed [due to small numbers in some of the cells].

**Conclusions:**

Our study revealed a significant, graded relationship between PSMU and poor SRH, highlighting the need to limit excessive social media use among adolescents to prevent health issues. The finding, that high family support may serve as a potential protective factor against poor SRH in adolescents with PSMU underscores the role of family engagement in safeguarding adolescent well-being.

**Supplementary Information:**

The online version contains supplementary material available at 10.1186/s12889-025-22927-6.

## Background

In recent decades, there has been a notable increase in social media usage among adolescents [1, 2]. These platforms offer young people the opportunity to create online public profiles, maintain connections with friends from their existing social networks, and interact with new people who share similar interests [3–5]. Social media also serves as a source of entertainment through its visual content, often boosting mood and inducing short-term positive feelings [6]. However, recent studies have highlighted potential negative effects associated with excessive social media use [7–9]. A key concern in this area is the concept of Problematic Social Media Use (PSMU), characterized by addiction-like symptoms such as excessive preoccupation, increased tolerance, withdrawal, mood modification (using social media to escape negative feelings), conflict, neglect of other activities, and difficulties in significant life areas [10, 11].

The inability to self-regulate social media use has been linked to a range of negative health outcomes, such as low life satisfaction, elevated levels of anxiety and depression, and an overall perception of poor health [8, 12–14]. However, fewer studies have examined potential protective factors in this context. To address this gap, our study investigates the association between PSMU and poor Self-Reported Health (SRH) in adolescents, as well as the potentially moderating (i.e., buffering) effect of family support. We chose SRH as our outcome measure because it captures current bodily, psychological, and somatic health issues while also being a well-established predictor of future health outcomes, including morbidity and mortality [15–16]. It is commonly used as a comprehensive measure of adolescent health (e.g [17–19]., 

PSMU may be associated with poor SRH through various mechanisms. Individuals who are unable to cope with their desire to engage in social media may be at increased risk for poor health due to the perception of lost control [10]. Excessive time spent on social media platforms may also imply a risk for poor health since it limits the time spent on health promoting habits such as adequate sleep, physical and social activities [13]. Moreover, adolescents experiencing negative emotions or stress may turn to social media platforms for support or relief. However, research suggest that the support obtained through these platforms may not be as effective as in-person social support. This implies that relying on social media to alleviate negative emotions or stress might not only be ineffective but could potentially exacerbate adverse consequences [20].

High family support has been found to promote adolescents’ well-being through the enhancement of resilience [20], assistance in emotional regulation, and elevation of positive feelings [21]. These factors may, in turn, reduce the risk of young people engageing in hazardous health behaviors and promote overall health [20]. Similarly, the stress and coping perspective, within social support theory, suggests that individuals with high social support may experience an improved balance between demands (stressors) and control (the ability to cope), which, in turn, increases the chances for positive emotions and good health [22, 23]. Consequently, a plausible hypothesis is that high family support may serve as a buffering factor– potentially reducing the incidence of poor SRH associated with PSMU. As such, it could be an important avenue for interventions aimed at promoting adolescent well-being in the context of increasing social media use.

However, a recent study by Lahti et al. [24], which analyzed data from the Health Behaviour in School-aged Children (HBSC) 2017/2018 survey in six European countries, found inconclusive evidence for this hypothesis. While cross-national analyses indicated a moderating effect of family support on the relationship between PSMU and SRH, national analyses showed that this effect was significant in only one of the six countries. This suggests that the possible buffering influence of family support on the adverse effects of PSMU on poor SRH may vary across different national contexts. Consequently, further research including additional countries is needed to increase our understanding of these relationships.

Additionally, gender, age, and family affluence have all been found to correlate with the risk of both PSMU and poor SRH among adolescents [24, 25]. Prevalence is generally higher among girls [8, 26], increases with age [8], and tends to be elevated among groups that are relatively more deprived [27, 28]. Therefore, in exploring the links between PSMU and poor SRH, it is essential to also consider the potential roles of gender, age, and family affluence.

### Aim of the study

The purpose of this study was to explore the relationship between Problematic Social Media Use (PSMU), poor Self-Reported Health (SRH), and family support among Swedish adolescents. More specifically, our aim was to determine the extent to which adolescents with PSMU tend to report poor SRH and whether high family support can buffer against any such association. The research questions addressed were:

1. Is there an association between higher levels of PSMU and poor SRH among Swedish adolescents, even after adjusting for factors such as gender, age, and family affluence?
2. Does high family support attenuate the strength of any potential association between PSMU and poor SRH?

## Methods

### Data materials

The data were derived from the 2017/2018 survey of the Swedish Health Behaviour in School-aged Children survey (HBSC, 2018). HBSC is a cross-national survey conducted every fourth year since 1983/1984 on behalf of the World Health Organisation (WHO). HBSC aims to evaluate the well-being, health behaviours, living conditions, and social interactions of boys and girls aged 11, 13, and 15. The questionnaire contains questions pertaining to topics such as subjective health status, alcohol and drug use, family and friend relationships, socioeconomic conditions, and health-related behaviours– including social media use. The questionnaires are administered by teachers and completed independently by the students in the class room [26, 29].

The 2017/18 Swedish HBSC survey employed a cluster sampling approach, initially selecting 450 schools and then randomly selecting one class per school. Ultimately, 213 out of 453 schools participated, corresponding to a drop-out rate of 53%. A total of 4,185 Swedish adolescents participated in the survey, comprising 1,181 11-year-olds, 1,452 13-year-olds, and 1,661 15-year-olds [30]. After excluding respondents with missing data on any of the study variables, the analytical sample was narrowed down to 3,274 individuals, representing 77.5% of the total sample.

### Measurements

PSMU was assessed using the Social Media Disorder Scale which assesses symptoms of PSMU through nine items (preoccupation, tolerance, withdrawal, persistence, escape, conflict, neglect of other activities, and difficulties in other life areas) with dichotomous (No/Yes) answers [11]. Internal consistency was high (Cronbach’s α = 0.75). Based on prior research, respondents with a sum score of 6 to 9 were identified as having PSMU. Those with a sum score between 2 and 5 were considered to be at moderate risk of PSMU, while a sum score of 0 to 1 indicated a low risk of PSMU [8, 31].

SRH was measured with the item: “Would you say your health is.?” with the response options: 1 = Excellent, 2 = Good, 3 = Fair, and 4 = Poor. These responses were then converted into a dichotomous variable categorized as: 0 = Good Health (combining Excellent and Good responses) and 1 = Less than Good Health (combining Fair and Poor responses). As demonstrated by Fosse and Haas [19] SRH can be considered a reliable and valid measure of adolescent health. It is stable from early and middle adolescence to young adulthood and effectively measures a range of physical and emotional dimensions of adolescent well-being.

Family support was assessed using three items from the Multidimensional Scale of Perceived Social Support (MSPSS) [32] tapping into emotional support: “My family really tries to help me”, “I receive the emotional help and support I need from my family”, and “I can discuss my problems with my family”. The same three items have been used to assess family support in a previous publication [33]. Responses to these statements were on a 7-point Likert-scale from 1 to 7, where 1 signifies “Very strongly disagree” and 7 denotes “Very strongly agree”. Internal consistency was high (Cronbach’s α = 0.87). These variables were aggregated into an additive index with the range 3–21 and subsequently recoded into a categorical variable with three levels, each encompassing (approximately) the same number of scale steps: Low family support (scores of 3–8), Moderate family support (scores of 9–14), and High family support (scores of 15–21).

Gender was categorized into two groups: boys and girls. No significant interaction between gender and PSMU in relation to poor SRH was found. Consequently, the analyses were performed using the combined sample of both boys and girls.

Age was divided into three groups: 11, 13, and 15 years old, corresponding to grades 5, 7, and 9 in the Swedish school system.

Family affluence was evaluated using the Family Affluence Scale, which considers the number of bathrooms, computers, and cars a family owns, whether the adolescent has their own bedroom, the presence of a dishwasher in the household, and the frequency of family vacations or travel [34]. Family affluence was then categorized into three levels based on relative measures: the lowest 20%, the middle 60%, and the highest 20%.

### Statistical analysis

First, descriptive statistics were explored. Following this, we assessed the distributions of the outcome variable (poor SRH) and the covariates (gender, age and family affluence), across the different categories of the exposure variable (PSMU). Chi-square tests were used to examine the associations between these variables. Next, to explore the association between PSMU and poor SRH, a series of logistic regression models were conducted. The results are reported as Odds Ratios (OR) with 95% confidence intervals (CI). Initial crude analyses included each independent variable individually. Model 1 then adjusted the main association for gender, age and family affluence. Model 2 added family support.

To investigate the potential moderating role of family support on the main association (Research Question 2), we conducted analyses to test for both multiplicative and additive interactions. The multiplicative interaction analysis tests whether the combined effect of two exposure variables exceeds the product of their individual effects—specifically, whether the effect of being exposed to both PSMU and low family support is greater than the effect of PSMU multiplied by the effect of low family support. In contrast, the additive interaction analysis assesses whether the combined effect of the two exposure variables exceeds the sum of their individual effects. Since significant effects may be found on one scale but not the other, including both approaches provide a more comprehensive assessment of how family support may moderate the main association [35].

Thus, model 3 introduced a multiplicative interaction term between PSMU and family support. The significance of the interaction was evaluated using a Wald test.

To assess a potential additive interaction between PSMU and family support on SHR, we constructed a joint exposure variable that stratified the three PSMU categories by the level of family support, resulting in a variable with nine categories. Additive interaction was then examined using Rothman’s formula for Relative Excess Risk due to Interaction (RERI = OR11 − OR10 − OR01 + 1). This formula quantifies the extent to which the combined effect of being exposed to both factors (PSMU and low family support) exceeds the sum of their individual effects. Using the Delta method, 95% CI: s were calculated, as well as the attributable proportion due to the interaction (AP = RERI/OR11) [35]. Following recommendations, we present our additive interaction analyses with a single common reference group [35], adolescents with low risk of PSMU and high family support.

To accommodate the hierarchical structure of the data (where students are nested within classes), we estimated robust standard errors for all models. The analyses were conducted using Stata version 17.0.

### Large Language models (LLMs)

ChatGPT, was used for grammar checking, proofreading the text, and offering clarifications.

## Results

Descriptive statistics of the study sample are presented in Table 1. the majority of adolescents (93.6%) reported good health, while 6.4% reported less than good health. Among the items used to measure PSMU, the highest prevalence was found for: “escape from negative feelings” (35.7%), “failed to spend less time on social media platforms” (31.0%), and “can’t think of anything else” (19.7%). The remaining items ranged between 8.2% and 14.8%. A majority of the adolescents (83.8%) were classified as having a low risk of PSMU, followed by 13.3% classified as having a moderate risk of PSMU, and 2.9% as having PSMU. Regarding family social support, 79.4% of the adolescents reported high support from their families, 14.4% reported moderate support, and 6.2% reported low support. The gender distribution was nearly equal, with 48.0% boys and 52.0% girls. Additionally, the age distribution within the study sample was 25.5% for 11-year-olds, 33.5% for 13-year-olds, and 41.0% for 15-year-olds. 13.9% of the sample belonged to the lowest family affluence category, 68.9% to the middle group, and 17.2% to the highest group. For more details about the full sample, including information on missing values, refer to Table S1 in the supplementary section.

**Table 1:** Descriptives of the study sample, *n* = 3 274

		*n*	%
**Self-rated health**			
Good health		3 159	93.6
Less than good health		208	6.4
**Social Media Disorder Scale items**			
1) Can’t think of anything else		644	19.7
2) Spend more time		482	14.7
3) Felt bad		484	14.8
4) Failed to spend less time		1 017	31.0
5) Neglected other activities		267	8.2
6) Arguments because of use		468	14.3
7) Lied about amount		398	12.1
8) Escape from negative feelings		1 169	35.7
9) Conflict with family because of use		323	9.9
**Problematic social media use (PSMU)**			
Low risk of PSMU		2 744	83.8
Moderate risk of PSMU		436	13.3
PSMU		94	2.9
**Family support**			
High support		2 599	79.4
Moderate support		471	14.4
Low support		204	6.2
**Gender**			
Boys		1 573	48.0
Girls		1 701	52.0
**Age**			
11 years		837	25.6
13 years		1 095	33.5
15 years		1 342	41.0
**Family affluence**			
Lowest 20ptc		456	13.9
Medium 60ptc		2 255	68.9
Highest 60ptc		563	17.2

Table 2 shows the distribution of the outcome and covariate variables across the categories of PSMU. The proportions of adolescents reporting less than good health increased significantly in a graded manner across the categories of PSMU (Chi square *p* < 0.001). Specifically, 5.0% of adolescents in the low-risk PSMU group reported less than good health, which increased to 12.6% among those at moderate risk, and further to 18.1% in the high-risk PSMU group.

**Table 2:** Distributions of outcome and covariates by exposure. Differences in the distributions by exposure groups assessed with Χ2 tests, *n* = 3 274

		Problematic social media use (PSMU) (%)			
		Low risk of PSMU	Moderate risk of PSMU	PSMU	x2
**Self-rated health**					
Less than good health		5.0	12.6	18.1	59.5***
**Family support**					
High support		87.0	11.0	2.0	
Moderate support		72.8	21.4	5.7	
Low support		69.6	23.5	6.9	95.1***
**Gender**					
Boys		88.8	9.2	2.0	
Girls		79.3	17.1	3.6	54.3***
**Age**					
11 years		88.7	9.3	2.0	
13 years		84.8	12.4	2.8	
15 years		80.0	16.5	3.4	29.4***
**Family affluence**					
Lowest 20ptc		81.1	16.0	2.9	
Medium 60ptc		84.3	12.9	2.8	
Highest 60ptc		84.2	12.8	3.0	3.4

***p < 0.001

The prevalence of PSMU demonstrated a significant negative gradient with respect to family support levels, increasing from 2.0% among adolescents with high support to 6.9% among those with low support (*p* < 0.001). However, no significant relationship was observed between family affluence and PSMU (*p* > 0.05).

Gender differences in PSMU prevalence were significant, with girls reporting higher rates of PSMU than boys (*p* < 0.001). Finally, a significant positive association was found between PSMU and age, with prevalence increasing from 2.0% among 11-year-olds to 3.4% among 15-year-olds (*p* < 0.001).

Results from the logistic regression analyses of PSMU and poor SRH are presented in Table 3. In the initial crude models, a statistically significant and graded association between PSMU and poor SRH was observed (p for trend < 0.001). Specifically, adolescents at moderate risk of PSMU were found to have 2.77 times higher odds of reporting less than good health (95% CI: 1.98–3.85), compared to those at low risk of PSMU. Adolescents with PSMU exhibited even greater odds, being 4.23 times more likely to report less than good health (95% CI: 2.45–7.31). Additionally, a positive correlation between age and poor SRH was identified, with 13-year-olds and 15-year-olds presenting 1.97 (95% CI: 1.22–3.17) and 2.58 (95% CI: 1.68–3.95) times higher odds of reporting less than good health, respectively. Girls were about twice as likely as boys to report less than good health (OR = 2.29, 95% CI 1.66–3.16). Adolescents in the mid category of family affluence were more likely to report less than good health compared to those in the lowest category, though no significant difference was found for adolescents in the highest category. A significant association was found between family support and poor SRH, where adolescents with low or moderate support reporting less than good health more frequently than those with high family support, with odds ratios of 6.03 (95% CI: 3.91–9.29) and 4.53 (95% CI: 3.29–6.23), respectively.

**Table 3:** Logistic regression analysis and multiplicative interaction: results of poor self-rated health regressed on problematic social media use (PSMU) and covariates. Odds ratios (OR) and 95% confidence intervals (95% CI) with robust standard errors. (*n* = 3 274)

		Crude		Model 1		Model 2		Model 3	
		OR	95% CI	OR	95% CI	OR	95% CI	OR	95% CI
**Problematic social media use**									
Low risk of PSMU (ref.)		1.00	-	1.00	-	1.00	-	1.00	-
Moderate risk of PSMU		2.77***	1.98, 3.85	2.32***	1.67, 3.22	1.84**	1.29, 2.63	2.45***	1.49, 4.03
PSMU		4.23***	2.45, 7.31	3.58***	2.09, 6.11	2.53**	1.44, 4.43	2.22	0.81, 6.14
**Gender**									
Boys (ref.)		1.00	-	1.00	-	1.00	-	1.00	-
Girls		2.29***	1.66, 3.16	2.00***	1.45, 2.70	1.93***	1.38, 2.67	1.93***	1.39, 2.69
**Age**									
11 years (ref.)		1.00	-	1.00	-	1.00	-	1.00	-
13 years		1.97**	1.22, 3.17	1.77**	1.09, 2.86	1.63*	1.09, 2.63	1.62*	1.01, 2.63
15 years		2.58***	1.68, 3.95	2.20***	1.43, 3.39	1.93**	1.24, 3.00	1.91**	1.23, 2.96
**Family affluence**									
Lowest 20ptc		1.62	0.93, 2.82	1.53	0.87, 2.69	1.24	0.71, 2.20	1.24	0.70, 2.19
Medium 60ptc		1.54*	1.02, 2.33	1.40	0.92, 2.15	1.28	0.82, 1,97	1.26	0.82, 1.95
Highest 60ptc (ref.)		1.00	-			1.00	-		
**Family support**									
High support (ref.)		1.00	-			1.00	-	1.00	-
Moderate support		4.53***	3.29, 6.23			3.74***	2.66, 5.26	4.37***	2.84, 6.71
Low support		6.03***	3.91, 9.29			4.74***	2.99, 7.51	5.05***	2.86, 8.95
**PSMU # Family support**									*p* = 0.61

***p < 0.001 **p < 0.01 *p < 0.05

Crude analyses include one independent variable at the time

Model 1 includes problematic social media use, age, gender and family affluence

Model 2 includes problematic social media use, age, gender, family affluence and family support

Model 3 includes problematic social media use, age, gender, family affluence, family support and interaction term

Adjusting for age, gender, and family affluence (Model 1) led to only minor attenuations of the association between PSMU and poor SRH (p for trend < 0.001). Further inclusion of family support (Model 2) resulted in additional attenuation of this association, which nonetheless remained significant (p for trend = 0.0013). The introduction of an interaction term between PSMU and family support in Model 3 did not achieve statistical significance (*p* = 0.61).

**Fig. 1:**
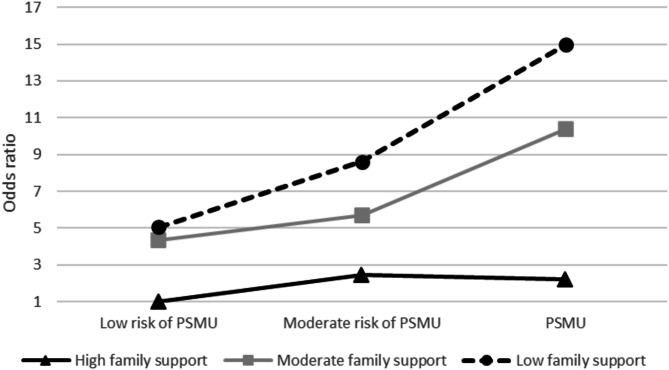
Odds ratios for poor SRH by a joint exposure variable (PSMU categories stratified by level of family support) adjusted for age, gender and family affluence. *n = 3 274.* Graph shows odds ratios for poor SRH among groups of adolescents with low, moderate and high family support, compared to the reference group; adolescents with low risk of PSMU and high family support

Results from the additive interaction analyses are reported in Table 4 and visualized in Fig. 1. Notably, the associations between PSMU and poor SRH appeared to differ across the categories of family support. The largest increase in the odds of reporting poor SRH with increasing levels of PSMU occurred among adolescents with low family support. Within this subgroup, those experiencing PSMU faced 15 times higher odds (OR = 14.96, 95% CI: 4.77–46.96) of reporting less than good health compared to the reference group, defined as adolescents with a low risk of PSMU and high family support. In contrast, the OR: s for adolescents with PSMU and moderate- or high family support were 10.39 (95% CI: 4.73–22.80) and 2.22 (95% CI: 0.80–6.14), respectively– thus, indicating a buffering effect of high family support among adolescents with PSMU. However, while calculation of the RERI and AP revealed that 8.68 (95% CI: -7.75-25.12) or 58% (95% CI: 0.10–1.05) of the odds in the double exposed group (adolescents with PSMU and low family support) was due to interaction, the estimate for RERI was not statistically significant (*p* = 0.301). This implies that, despite suggestive trends, the additive interaction between PSMU and lack of family support on poor SRH could not be statistically confirmed.

**Table 4:** Additive interaction analysis, results from logistic regression on poor SRH. Table includes a joint exposure variable (PSMU categories stratified by level of family support), odds ratios (OR), 95% confidence intervals (95% CI). *n = 3 274*

	OR	95% CI
**Problematic social media use stratified by level of family support**		
Low risk of PSMU/High family support (ref.)	1	-
Moderate risk of PSMU/High family support	2.45***	1.49, 4.03
PSMU/ High family support	2.22	0.80, 6.14
Low risk of PSMU/Moderate family support	4.37***	2.85, 6.71
Moderate risk of PSMU/Moderate family support	5.68***	3.29, 9.79
PSMU/Moderate family support	10.39***	4.73, 22.80
Low risk of PSMU/ Low family support	5.05***	2.85, 8.95
Moderate risk of PSMU/ Low family support	8.61***	4.31, 17.19
PSMU/ Low family support	14.96***	4.77, 46.96

***p < 0.001 **p < 0.01 *p < 0.05

Adjusted for age, gender and relative family affluence

## Discussion

Our study explored the links between PSMU, SRH and family support among Swedish adolescents. More specifically, we aimed to determine whether adolescents with PSMU have an increased risk of poor SRH, and whether high family support can buffer against any such association.

Our first research question addressed whether there was an association between PSMU and poor SRH among Swedish adolescents, even after adjusting for gender, age and family affluence. After adjustment for these covariates, our findings revealed a clear, graded association between PSMU and poor SRH, with adolescents classified as having PSMU showing the highest odds for poor SRH, followed by those at moderate risk for PSMU. These results align with prior studies from Canada [31], Finland [8], and various European countries [24], confirming that the association between PSMU and poor SRH also holds true within the Swedish adolescent population.

The second research question explored the potential moderating role of family support in the association between PSMU and poor SRH. Our analyses provided only suggestive evidence of such moderation. While no effect modification was found in the multiplicative interaction analyses, the additive interaction analyses indicated that the association between PSMU and poor SRH varies based on the level of family support. Specifically, high family support appeared to buffer against the negative impact of PSMU on SRH, as evidenced by the lack of a significant association between PSMU and poor SRH among adolescents with strong family support. Conversely, for adolescents with low family support, PSMU was linked to a nearly 15-fold increase in the odds of poor SRH, with an attributable proportion (AP) due to interaction of 58%. Still, the small size of this group (*n = 14*) resulted in a non-significant relative excess risk due to interaction (RERI) estimate, preventing us from drawing any definite conclusions about the role of family support in this context. Our inconclusive results align with the study by Lahti et al. [24], who examined these associations in HBSC data from six European countries and reported a moderating effect of family support on the PSMU-SRH relationship in cross-national analyses, but only in one of the six countries in national analyses. It is important to note that Lahti et al. tested only for multiplicative interaction, and it is possible that incorporating measures of additive interaction could have yielded more significant results. Still, our findings, along with those of Lahti et al. [24]., suggest a potential protective effect of high family support against poor SRH in adolescents with PSMU. Additionally, the small size of this group underscores the need for future studies based on larger or cross-national pooled samples, including measures of both multiplicative and additive interaction to fully confirm this association.

Our findings are in line with the stress and coping framework, which suggests that high social support may enhance coping with stress by improving the balance between stressors and coping abilities [23]. Specifically, strong family support may help provide adolescents with strategies that are effective for managing different life stressors, thus protecting against the possible negative health impacts of PSMU.

Given the associations between PSMU and poor SRH, it is also important to note that the distribution of PSMU varies by gender and age. Specifically, higher risks of PSMU were observed among girls compared to boys and among older adolescents compared to younger ones. Notably, however, no statistically significant differences were found based on family affluence in the fully adjusted model.

### Strengths and limitations

Our study’s strength lies in its broad national coverage of Swedish adolescents, employing validated measures for PSMU, SRH, and family support. Non-response analyses from a previous round of the Swedish HBSC study show that the non-response was not systematic among the sampled schools. If this also holds true for the 2017/18 Swedish HBSC survey, our results can be considered generalizable to the surveyed youth population in Sweden [30]. However, a limitation of our study is the challenge of attaining adequate statistical power for different categories within the combined variable used for additive interaction, due to the small number of participants in certain categories. Additionally, the cross-sectional dataset limits our ability to establish causality between PSMU and SRH [36]. While PSMU may contribute to poor SRH, it is also plausible that some individuals engage in excessive social media use as a coping mechanism for existing physical or mental health challenges [37]. Another limitation is the omission of potentially confounding variables that could affect the risk for both PSMU and poor SRH [38]. For example, overlapping experiences of PSMU and poor SRH among some students could stem from underlying mental health issues or substance use disorders. Another concern is the potential for non-response bias at the respondent level; Some adolescents selected for the study may have chosen not to participate, failed to answer key questions, or were absent on the survey day, possibly due to poor SRH or PSMU. Despite these limitations, it is more probable that this potential bias has resulted in an underestimation, rather than an overestimation, of the primary association under investigation.

### Recommendations for future research

The findings of this study, alongside prior research, highlight the need to further explore the effects of PSMU on adolescent health, with particular attention to the potential protective role of family support. To enhance the generalizability of these findings, future research should examine a broader range of health outcomes. This includes not only various valid and reliable indicators of subjective health and well-being, including those based on single as well as multiple items (e.g., psychosomatic complaints, life satisfaction, and mental well-being) but also more severe mental health conditions (e.g., the need for psychiatric treatment), while investigating how family support may moderate these relationships. Furthermore, to achieve a thorough understanding of the temporal dynamics and to evaluate both short- and long-term effects, future studies should incorporate longitudinal data.

### Implications of the findings

The findings of the study enhance our understanding of the association between PSMU and poor SRH among adolescents and carry important policy implications. First, the significant and graded association between PSMU and poor SRH underscores the need for public health strategies that curtail excessive or problematic social media use to help prevent health issues in this group. This could include universal interventions to raise awareness among adolescents and their families about the risks associated with PSMU. Second, the study points to family support as a potential protective factor for adolescents with PSMU, indicating the need for programs that enhance parental caregiving and supportive skills and emphasize the importance of their involvement in their children’s wellbeing [39]. This approach advocates for targeted public health strategies focused on more vulnerable adolescent populations, alongside strengthening family support systems, to effectively address the impacts of PSMU on adolescent health.

## Conclusions

Our study demonstrated that PSMU is significantly associated with poor SRH in a graded manner. These findings emphasize the importance of reducing excessive or problematic social media use among adolescents to prevent health problems within this group. The observation of a potentially protective effect of high family support on the risk for poor SRH among adolescents with PSMU accentuates the significance of family involvement in ensuring adolescents’ well-being in this context. Future research, utilizing larger or cross-national pooled samples and including measures of both multiplicative and additive interaction, is essential to fully validate these associations.

## Electronic supplementary material

Below is the link to the electronic supplementary material.

Supplementary Material 1

## Data Availability

Data are available from the HBSC Data Management Center, University of Bergen. Please see:https://www.uib.no/en/hbscdata/113290/open-access

## Abbreviations

95% CI: 95% Confidence Interval
AP: Attributable Proportion
HBSC: Health Behaviour in School-aged Children
OR: Odds Ratio
PSMU: Problematic Social Media Use
RERI: Relative Excessive Risk due to Interaction
SRH: Self Rated Health
WHO: World Health Organization

## References

[1] Delisle Nyström C, Carlander A, Cassel S, Rosell M, J-Son Höök M, Löf M. Physical activity and screen time in Swedish children and adolescents: the generation Pep study 2018–2021. Acta Paediatr. 2023;112(3):460–8. 36371645 10.1111/apa.16594 PMC10098717

[2] Vannucci A, Simpson EG, Gagnon S, Ohannessian CM. Social media use and risky behaviors in adolescents: A meta-analysis. J Adolesc. 2020;79:258–74. 32018149 10.1016/j.adolescence.2020.01.014

[3] Xuan YJ, Amat MAC. Social media addiction and young people: A systematic review of literature. J Crit Rev. 2020;7(13):537–41.

[4] Ryan T, Allen KA, Gray DL, McInerney DM. How social are social media? A review of online social behaviour and connectedness. J Relationships Res. 2017;8:e8.

[5] Savci M, Akat M, Ercengiz M, Griffiths MD, Aysan F. Problematic social media use and social connectedness in adolescence: the mediating and moderating role of family life satisfaction. Int J Mental Health Addict 2020:1–17.

[6] Smith T, Short A. Needs affordance as a key factor in likelihood of problematic social media use: validation, latent profile analysis and comparison of TikTok and Facebook problematic use measures. Addict Behav. 2022;129:107259. 35091200 10.1016/j.addbeh.2022.107259

[7] Marttila E, Koivula A, Räsänen P. Does excessive social media use decrease subjective well-being? A longitudinal analysis of the relationship between problematic use, loneliness and life satisfaction. Telematics Inform. 2021;59:101556.

[8] Paakkari L, Tynjälä J, Lahti H, Ojala K, Lyyra N. Problematic social media use and health among adolescents. Int J Environ Res Public Health. 2021;18(4):1885. 33672074 10.3390/ijerph18041885 PMC7919645

[9] Jin L, Hao Z, Huang J, Akram HR, Saeed MF, Ma H. Depression and anxiety symptoms are associated with problematic smartphone use under the COVID-19 epidemic: the mediation models. Child Youth Serv Rev. 2021;121:105875. 36540404 10.1016/j.childyouth.2020.105875 PMC9756353

[10] Boer M, Van Den Eijnden RJ, Boniel-Nissim M, Wong S-L, Inchley JC, Badura P, Craig WM, Gobina I, Kleszczewska D, Klanšček HJ. Adolescents’ intense and problematic social media use and their well-being in 29 countries. J Adolesc Health. 2020;66(6):S89–99. 32446614 10.1016/j.jadohealth.2020.02.014 PMC7427320

[11] Boer M, van den Eijnden RJ, Finkenauer C, Boniel-Nissim M, Marino C, Inchley J, Cosma A, Paakkari L, Stevens GW. Cross‐national validation of the social media disorder scale: findings from adolescents from 44 countries. Addiction. 2022;117(3):784–95. 34605094 10.1111/add.15709 PMC7614030

[12] Brailovskaia J, Truskauskaite-Kuneviciene I, Kazlauskas E, Margraf J. The patterns of problematic social media use (SMU) and their relationship with online flow, life satisfaction, depression, anxiety and stress symptoms in Lithuania and in Germany. Curr Psychol 2021:1–12.

[13] Raudsepp L, Kais K. Longitudinal associations between problematic social media use and depressive symptoms in adolescent girls. Prev Med Rep. 2019;15:100925. 31304081 10.1016/j.pmedr.2019.100925 PMC6603436

[14] Stockdale LA, Coyne SM. Bored and online: reasons for using social media, problematic social networking site use, and behavioral outcomes across the transition from adolescence to emerging adulthood. J Adolesc. 2020;79:173–83. 31978836 10.1016/j.adolescence.2020.01.010

[15] Jylhä M. What is self-rated health and why does it predict mortality? Towards a unified conceptual model. Soc Sci Med. 2009;69(3):307–16. 19520474 10.1016/j.socscimed.2009.05.013

[16] Vie TL, Hufthammer KO, Holmen TL, Meland E, Breidablik HJ. Is self-rated health in adolescence a predictor of prescribed medication in adulthood? Findings from the Nord Trøndelag health study and the Norwegian prescription database. SSM-Population Health. 2018;4:144–52. 29349283 10.1016/j.ssmph.2017.11.010 PMC5769112

[17] Potrebny T, Torsheim T, Due P, Välimaa R, Suominen S, Eriksson C. Trends in excellent self-rated health among adolescents: a comparative nordic study. Välfärdsforskning|Nordisk välfärdsforskning| Nordic Welf Res. 2019;4(2):67–76.

[18] Marques A, Peralta M, Santos T, Martins J, de Matos MG. Self-rated health and health-related quality of life are related with adolescents’ healthy lifestyle. Public Health. 2019;170:89–94. 30978580 10.1016/j.puhe.2019.02.022

[19] Fosse NE, Haas SA. Validity and stability of self-reported health among adolescents in a longitudinal, nationally representative survey. Pediatrics. 2009;123(3):e496–501. 19254984 10.1542/peds.2008-1552

[20] Lin S, Yuan Z, Niu G, Fan C, Hao X. Family matters more than friends on problematic social media use among adolescents: mediating roles of resilience and loneliness. Int J Mental Health Addict 2023:1–19. 10.1007/s11469-023-01026-w PMC9933806 36811077

[21] Morris AS, Criss MM, Silk JS, Houltberg BJ. The impact of parenting on emotion regulation during childhood and adolescence. Child Dev Perspect. 2017;11(4):233–8.

[22] Lakey B, Cohen S. Social support and theory. *Social support measurement and intervention: A guide for health and social scientists* 2000, 29:29–49.

[23] Shi X, Wang J, Zou H. Family functioning and internet addiction among Chinese adolescents: the mediating roles of self-esteem and loneliness. Comput Hum Behav. 2017;76:201–10.

[24] Lahti H, Kulmala M, Hietajärvi L, Lyyra N, Kleszczewska D, Boniel-Nissim M, Furstova J, van den Eijnden R, Sudeck G, Paakkari L. What counteracts problematic social media use in adolescence? A cross-national observational study. J Adolesc Health. 2024;74(1):98–112. 37777950 10.1016/j.jadohealth.2023.07.026

[25] Jerdén L, Burell G, Stenlund H, Weinehall L, Bergström E. Gender differences and predictors of self-rated health development among Swedish adolescents. J Adolesc Health. 2011;48(2):143–50. 21257112 10.1016/j.jadohealth.2010.06.005

[26] Inchley J, Currie D, Budisavljevic S, Torsheim T, Jåstad A, Cosma A, Kelly C, Arnarsson A, Samdal O. Spotlight on adolescent health and well-being. Findings from the 2017/2018 health behaviour in school-aged children (HBSC) survey in Europe and Canada. International report. Volume 1. Key findings. Copenhagen: WHO Regional Office for Europe; 2020. *Contract No: Licence: CC BY-NC-SA* 2020, 3.

[27] Lenzi M, Elgar FJ, Marino C, Canale N, Vieno A, Berchialla P, Stevens GW, Boniel-Nissim M, van den Eijnden RJ, Lyyra N. Can an equal world reduce problematic social media use? Evidence from the health behavior in School-aged children study in 43 countries. Inform Communication Soc. 2023;26(14):2753–74.

[28] Currie C, Molcho M, Boyce W, Holstein B, Torsheim T, Richter M. Researching health inequalities in adolescents: the development of the health behaviour in School-Aged children (HBSC) family affluence scale. Soc Sci Med. 2008;66(6):1429–36. 18179852 10.1016/j.socscimed.2007.11.024

[29] Moor I, Winter K, Bilz L, Bucksch J, Finne E, John N, Kolip P, Paulsen L, Ravens-Sieberer U, Schlattmann M. The 2017/18 health behaviour in School-aged children (HBSC) study–Methodology of the world health organization’s child and adolescent health study. J Health Monit. 2020;5(3):88. 35146275 10.25646/6904 PMC8734187

[30] The Swedish Public Health Agency. Skolbarns hälsovanor i Sverige 2017/18. Grundrapport (Schoolchildren’s Health Habits in Sweden 2017/18. Basic Report). Folkhälsomyndigheten, 2018.

[31] Craig W, Gariepy G, Mayne K, Atallah R, Georgiades K. Mental health and problematic social media use in Canadian adolescents findings from the 2018 health behaviour of School-aged children (HBSC) study. In. Public Health Agency of Canada, Ottawa; 2021.

[32] Zimet GD, Dahlem NW, Zimet SG, Farley GK. The multidimensional scale of perceived social support. J Pers Assess. 1988;52(1):30–41. 10.1080/00223891.1990.9674095 2280326

[33] Venäläinen J, Låftman SB, Landberg J. Weight status and psychosomatic complaints in Swedish adolescent boys and girls: does family support play a buffering role? BMC Public Health. 2024;24(1):3024. 39482648 10.1186/s12889-024-20517-6 PMC11529216

[34] Torsheim T, Cavallo F, Levin KA, Schnohr C, Mazur J, Niclasen B, Currie C, Group FDS. Psychometric validation of the revised family affluence scale: a latent variable approach. Child Indic Res. 2016;9:771–84. 27489572 10.1007/s12187-015-9339-x PMC4958120

[35] VanderWeele TJ, Knol MJ. A tutorial on interaction. Epidemiol Methods. 2014;3(1):33–72.

[36] Wang X, Cheng Z. Cross-sectional studies: strengths, weaknesses, and recommendations. Chest. 2020;158(1):S65–71. 32658654 10.1016/j.chest.2020.03.012

[37] Houghton S, Lawrence D, Hunter SC, Rosenberg M, Zadow C, Wood L, Shilton T. Reciprocal relationships between trajectories of depressive symptoms and screen media use during adolescence. J Youth Adolesc. 2018;47:2453–67. 30046970 10.1007/s10964-018-0901-y PMC6208639

[38] Brunborg GS, Andreas JB. Increase in time spent on social media is associated with modest increase in depression, conduct problems, and episodic heavy drinking. J Adolesc. 2019;74:201–9. 31254779 10.1016/j.adolescence.2019.06.013

[39] Alfredsson EK, Thorvaldsson V, Axberg U, Broberg AG. Parenting programs during adolescence: outcomes from universal and targeted interventions offered in real-world settings. Scand J Psychol. 2018;59(4):378–91. 29697869 10.1111/sjop.12446

