# Supplementary material for: Problematic social media use and self-rated health among Swedish adolescents: is the association moderated by family support?
Source: BMC Public Health. 2025 May 6;25:1670. doi: 10.1186/s12889-025-22927-6 (PMC12054090; doi:10.1186/s12889-025-22927-6)
Supplement: Supplementary file 1 — Supplementary Material 1 [file 12889_2025_22927_MOESM1_ESM.docx]

Supplementary materials

**Table S1**. Descriptives of the full sample, *n*= 4 185

|  |  |  | |  |  | | *n* | | | % | |
| --- | --- | --- | --- | --- | --- | --- | --- | --- | --- | --- | --- |
| **Self-rated health** | | | |  |  | |  | | |  | |
|  |  | Good health | |  |  | | 3.841 | | | 93.3% | |
|  |  | Less than good health | |  |  | | 276 | | | 6.7% | |
|  |  | *Missing* | |  |  | | *68* | | | - | |
| **Social Media Disorder Scale items** | | | | |  | |  | | |  | |
|  | 1) Can't think of anything else | | | |  | |  | | |  | |
|  |  | No | |  |  | | 2 922 | | | 79.8% | |
|  |  | Yes | |  |  | | 742 | | | 20.2% | |
|  |  | *Missing* | |  |  | | *521* | | | - | |
|  | 2) Spend more time | | |  |  | |  | | |  | |
|  |  | No | |  |  | | 3 094 | | | 84.4% | |
|  |  | Yes | |  |  | | 573 | | | 15.6% | |
|  |  | *Missing* | |  |  | | *218* | | | - | |
|  | 3) Felt bad | | |  |  | |  | | |  | |
|  |  | No | |  |  | | 3 096 | | | 85.0% | |
|  |  | Yes | |  |  | | 548 | | | 15.0% | |
|  |  | *Missing* | |  |  | | *541* | | | - | |
|  | 4) Failed to spend less time | | | |  | |  | | |  | |
|  |  | No | |  |  | | 2 496 | | | 68.1% | |
|  |  | Yes | |  |  | | 1168 | | | 31.9% | |
|  |  | *Missing* | |  |  | | *521* | | | - | |
|  | 5) Neglected other activities | | | |  | |  | | |  | |
|  |  | No | |  |  | | 3 340 | | | 91.4% | |
|  |  | Yes | |  |  | | 314 | | | 8.6% | |
|  |  | *Missing* | |  |  | | *531* | | | - | |
|  | 6) Arguments because of use | | | |  | |  | | |  | |
|  |  | No | |  |  | | 3 112 | | | 85.3% | |
|  |  | Yes | |  |  | | 535 | | | 14.7% | |
|  |  | *Missing* | |  |  | | *538* | | | - | |
|  | 7) Lied about amount | | | |  | |  | | |  | |
|  |  | No | |  |  | | 3 197 | | | 87.3% | |
|  |  | Yes | |  |  | | 466 | | | 12.7% | |
|  |  | *Missing* | |  |  | | *522* | | | - | |
|  | 8) Escape from negative feelings | | | | | |  | | |  | |
|  |  | No | |  |  | | 2 341 | | | 64.1% | |
|  |  | Yes | |  |  | | 1 311 | | | 35.9% | |
|  |  | *Missing* | |  |  | | *533* | | | - | |
|  | 9) Conflict with family because of use | | | | | |  | | |  | |
|  |  | No | |  |  | | 3 277 | | | 89.7% | |
|  |  | Yes | |  |  | | 376 | | | 10.3% | |
|  |  | *Missing* | |  |  | | *532* | | | - | |
| **Problematic social media use (PSMU)** | | | | | |  | |  |  | |  |
|  | | Low risk of PSMU | | | |  | |  | 2 931 | | 83.7% |
|  | | Moderate risk of PSMU | | | |  | |  | 469 | | 13.4% |
|  | | PSMU | | | |  | |  | 103 | | 2.5% |
|  | | *Missing* |  | | |  | |  | *682* | | - |
| **Family support** | | | | | | | |  |  | |  |
|  | | High support | | | |  | |  | 3 172 | | 79.2% |
|  | | Moderate support | | | | | |  | 574 | | 14.3% |
|  | | Low support | | | |  | |  | 261 | | 6.5% |
|  | | *Missing* |  | | |  | |  | *178* | | - |
| **Gender** | |  |  | | |  | |  |  | |  |
|  | | Boys |  | | |  | |  | 2 081 | | 49.7% |
|  | | Girls |  | | |  | |  | 2 104 | | 50.3% |
|  | | *Missing* |  | | |  | |  | - | | - |
| **Age** | |  |  | | |  | |  |  | |  |
|  | | 11 years |  | | |  | |  | 1 174 | | 28.1% |
|  | | 13 years |  | | |  | |  | 1 423 | | 34.0% |
|  | | 15 years |  | | |  | |  | 1 588 | | 38.0% |
|  | | *Missing* |  | | |  | |  | - | | - |
| **Family affluence** | | | | | |  | |  |  | |  |
|  | | Lowest 20ptc | | | |  | |  | 628 | | 15.7 |
|  | | Medium 60ptc | | | |  | |  | 2,688 | | 67.0 |
|  | | Highest 60ptc |  | | |  | |  | 696 | | 17.3 |
|  | | *Missing* |  | | |  | |  | *173* | | *-* |
